# Enhancing the precision and uniformity of clinical target volume delineation for cervical cancer through a structured educational program

**DOI:** 10.1186/s12909-026-09203-w

**Published:** 2026-04-13

**Authors:** Zhaoqi Gu, Yulin Liu, Yongguang Liang, Xin Lian, Fuquan Zhang, Jie Qiu, Bo Yang, Shuai Sun, Xiaorong Hou

**Affiliations:** 1 Department of Radiation Oncology, Peking Union Medical College Hospital, Chinese Academy of Medical Sciences & Peking Union Medical College, No. 1 Shuaifuyuan, Wangfujing, Dongcheng District, Beijing, 100730 China; 2 Eight-Year Medical Doctor Program, Chinese Academy of Medical Sciences and Peking Union Medical College, Beijing, China

**Keywords:** Cervical cancer, Radiotherapy, Clinical target volume, Delineation, Interobserver variability, Continuing Medical Education

## Abstract

**Purpose:**

To evaluate the short-term impact of a structured educational program on the accuracy and consistency of clinical target volume (CTV) delineation for external beam radiotherapy (EBRT) in cervical cancer among Chinese radiation oncologists.

**Method:**

A total of 45 radiation oncologists participated in a multi-component training course covering guideline-based principles of cervical cancer EBRT, case-based contouring exercises, expert-led reviews, and live demonstrations. Participants performed pre- and post-training delineations on two representative cases (definitive and postoperative radiotherapy) and completed corresponding questionnaires. Delineation quality was assessed using spatial overlap, boundary accuracy, and interobserver variability metrics, with two automated systems included for performance ranking. Correlations between subjective self-assessments and objective metrics were analyzed.

**Results:**

Significant post-training improvements were observed in both subjective confidence and objective contouring performance. The primary outcomes, Dice Similarity Coefficient (DSC) and Average Surface Distance (ASD), improved significantly in both clinical cases (*p* < 0.05), together with several secondary geometric metrics. Interobserver variability decreased across multiple indices. Exploratory analyses suggested alignment between subjective self-assessment and objective contouring quality after training, while more modest improvements were observed in anatomically complex subregions.

**Conclusions:**

This study demonstrates that a structured educational intervention significantly enhances the accuracy, consistency, and self-perceived competency of cervical cancer target delineation among radiation oncologists. These findings support the integration of such programs into routine professional development to standardize radiotherapy planning and improve clinical outcomes.

**Supplementary Information:**

The online version contains supplementary material available at 10.1186/s12909-026-09203-w.

## Take-Home Points

- A targeted educational program significantly improved the accuracy and consistency of Clinical Target Volume (CTV) delineation for cervical cancer.
- The program not only boosted participants’ self-rated confidence and proficiency but also strengthened the correlation between their subjective scores and objective delineation quality.
- While overall delineation improved, gains were less pronounced in challenging anatomical areas like the para-aortic and pelvic nodal regions. This finding suggests that future training should incorporate specialized modules focused on these specific subregions to achieve greater precision.
- Although automated segmentation systems provide a high-performance benchmark, the training successfully narrowed the performance gap between clinicians and these AI tools.

## Introduction

Cervical cancer ranks as the fourth most common cancer diagnosed and the fourth leading cause of cancer-related deaths in women [1]. Improvements in radiotherapy technology have led to reduced treatment-related toxicity for women with locally-advanced disease [2]. The standard treatment for locally advanced cervical cancer (stages IB2–IVA) is a combination of external beam radiotherapy (EBRT) to the pelvis and intracavitary brachytherapy (ICBT) [3]. The effectiveness of radiotherapy heavily relies on the accurate delineation of the Clinical Target Volume (CTV) as it directly influences the radiation dose delivered to the tumor and surrounding tissues. Accurate target definition ensures that the maximum dose is applied to cancer cells while minimizing exposure to adjacent healthy tissues, thereby reducing the risk of both acute and long-term toxicities. Furthermore, uniformity in delineation practices among clinicians is essential to achieve consistent treatment outcomes. Variability in delineation can lead to differences in treatment efficacy and patient side effects.

Despite the existence of standard guidelines, intra- and interobserver variability remains a significant challenge in planning radiotherapy [4]. Studies have documented discrepancies in contouring practices [5, 6], which can lead to inconsistencies in radiation dose distribution and potentially compromise treatment outcomes. Existing literature has summarized several categories of interventions to reduce interobserver variability (IOV), including guideline-based protocols, educational and feedback-based training, autocontouring tools, and multimodal imaging approaches [7]. Among these, structured training programs—including online digital platform [7], teaching lectures [8], interactive teaching session [9] and intensive boot camp [10]—have been shown to effectively reduce these discrepancies [11]. Evidence from clinical practice has also demonstrated that such structured interventions can reduce interobserver variability in real-world settings [12]. In addition, emerging AI-based automatic segmentation systems have shown potential in reducing contouring variability, although human oversight remains crucial to ensure clinical accuracy [13]. Given the existing variability in target delineation and the current absence of studies on educational interventions within the scope of cervical cancer target delineation, we conducted this study to investigate the short-term effects of a targeted educational program on the accuracy of clinical target volume (CTV) delineation for EBRT in cervical cancer among Chinese radiation oncologists. This study is among the first to apply both subjective and objective measures to evaluate the effect of an educational intervention on CTV delineation in cervical cancer radiotherapy. We aimed to examine whether structured training could improve the accuracy and consistency of target contouring among radiation oncologists, while also exploring its relationship with emerging automatic segmentation tools.

## Methods

### Educational program design

The course included a lecture on EBRT for cervical cancer, followed by another session on cervical cancer external beam radiation target delineation. This educational program consisted of four components. First, a lecture on EBRT for cervical cancer was conducted, reviewing consensus guidelines for cervical cancer target delineation and the latest research. Second, clinical information of two representative cases was presented, with each participant performing initial delineation of CTV (Clinical Target Volume) and OAR (Organs at Risk) according to target delineation requirements. Third, an experienced physician demonstrated and explained common delineation errors in these two typical cases, highlighting differences between standard target volumes and problematic target volumes. Clinical target volumes (CTV) were delineated in accordance with Radiation Therapy Oncology Group consensus guidelines [14], including uterine corpus (CTV-U), gross tumor, vaginal tissues, uterine cervix, parametria (CTV-C), common, internal and external iliac, obturator, and presacral lymph nodes (CTV-N) [15]. The reference contours (ground truth) used for evaluating participant performance were initially generated by a senior radiation oncologist at our center with over 20 years of experience in cervical cancer radiotherapy. These contours were subsequently reviewed and finalized through a departmental consensus meeting involving three additional senior radiation oncology experts. The final consensus contours served as the reference standard for all quantitative analyses.

Fourth, a live demonstration of standard target delineation was conducted, emphasizing the rationale behind target delineation approaches for both clinical scenarios. Finally, after training completion, participants were required to repeat target delineation on the same CT images. Participants completed pre- and post-training questionnaires to assess their familiarity and confidence regarding cervical cancer diagnosis, treatment principles, and EBRT target delineation. All questionnaire items used a 5-point Likert scale; higher scores indicated greater confidence/proficiency or, for the concern item, reduced worry. The questionnaires are provided in the Supplementary Materials.

### Patient characteristics

The training program featured two representative cases:

- Case 1 (Definitive radiotherapy): A 55-year-old woman with stage IIIC1r (FIGO 2018) cervical squamous cell carcinoma. Pelvic MRI demonstrated a 24 × 18 mm cervical mass extending beyond the external os (lower 1/3 vagina spared) and left pelvic lymphadenopathy (short axis 13 mm).
- Case 2 (Postoperative radiotherapy): A 52-year-old woman with stage IIIC1p HPV-associated poorly differentiated invasive cervical adenocarcinoma, 1 month post-radical hysterectomy. Pathology showed outer 1/3 cervical stromal invasion with lymphovascular space involvement and right presacral lymph node metastasis. The patient received 2 cycles of adjuvant paclitaxel/carboplatin chemotherapy.

Both cases represent common clinical scenarios in cervical cancer radiotherapy. Imaging studies (contrast-enhanced CT and MRI from L2-L3 junction to proximal femur, 5 mm slice thickness) were acquired in supine position with standardized bladder/rectal preparation. Images were transferred to the Varian treatment planning system. Participants reviewed anonymized clinical data including imaging findings through standardized PowerPoint presentations. All radiation oncologists independently delineated CTV on CT images based on clinical requirements and institutional protocols.

### Participants

A total of 45 radiation oncologists participated in this program. Among all participants, 22 held senior titles (chief or associate chief physicians), while 23 held junior titles (attending or resident physicians). The average professional experience was 12.18 years, and prior to the program, each physician had managed an average of 76.59 gynecological radiation therapy patients.

**Fig. 1:**
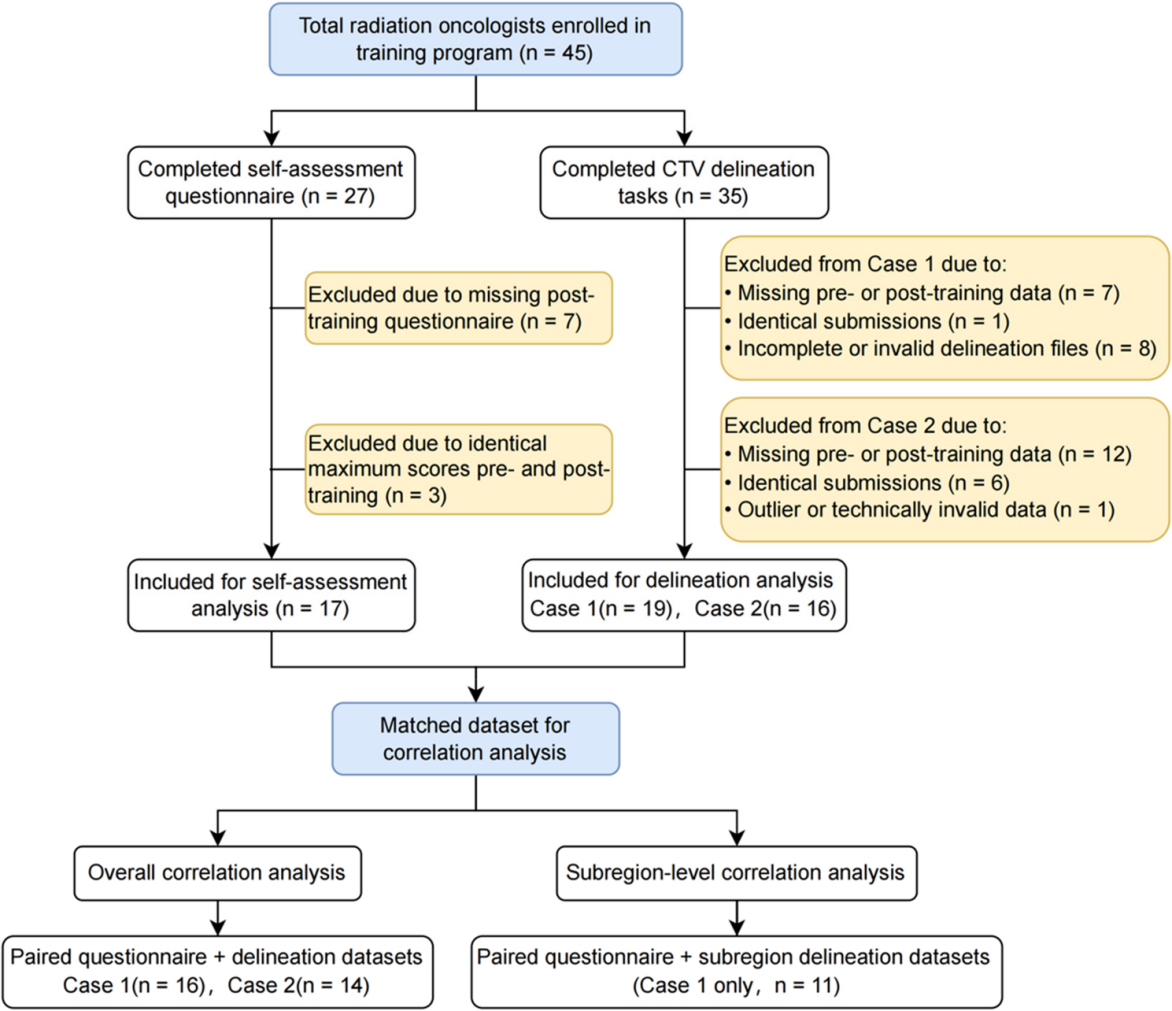
Participant inclusion flowchart for self-assessment and contouring analysis

Of the 45 radiation oncologists enrolled, participation in the delineation and questionnaire tasks was voluntary; 27 completed the self-assessment questionnaire, and 35 completed the CTV delineation tasks. After exclusion based on predefined quality control criteria — including missing post-training data, identical pre- and post-training submissions, ceiling effects in questionnaire responses, and incomplete or technically invalid delineation files — 17 participants were retained for self-assessment analysis, and 19/16 for Case 1 and Case 2 delineation analyses, respectively. A total of 30 participants had paired questionnaire and delineation data, and 11 of them provided valid subregion-level delineation in Case 1. The full inclusion and exclusion process is illustrated in Fig. 1.

### Target volume delineation

The training program established case-specific target delineation protocols. For Case 1 (definitive radiotherapy), participants were required to delineate the pelvic nodal clinical target volume (CTV-Np, encompassing internal iliac, obturator, common iliac, external iliac, and presacral regions), para-aortic nodal CTV (CTV-Na), uterine corpus CTV (CTV-U), cervical tumor CTV (CTV-C including adjacent parametrial/vaginal involvement), and organs at risk (OARs: bladder, rectum, small bowel, femoral heads, bone marrow, and spinal cord). This structured approach allowed systematic evaluation of anatomical comprehension despite clinical practice typically consolidating these regions. For Case 2 (postoperative radiotherapy), delineation focused on the surgical bed CTV (vaginal cuff and pelvic nodal regions) with identical OAR prioritization. Critical abdominal organs (kidney/liver/stomach/duodenum) were excluded from OAR delineation per institutional protocols, reflecting clinical relevance in cervical cancer radiotherapy planning.

### Statistical analysis

All data underwent rigorous screening to ensure validity. Self-assessment scores obtained from the questionnaires were reported as mean ± standard deviation (SD), with paired comparisons between pre- and post-training using the Wilcoxon signed-rank test.

Contouring quality was assessed by comparing pre- and post-training delineations (Vpre, Vpost) with expert-defined reference volumes (Vref). As summarized in Table S1, contouring quality metrics were grouped into two categories: spatial overlap and consistency metrics (Dice Similarity Coefficient [DSC], Conformity Index [CI], Inclusion Index [Inclusion], Relative Volume Difference [RVD]) and geometric boundary accuracy metrics (Average Surface Distance [ASD], Distance between Centers of Mass [DC], 95th percentile Hausdorff Distance [95%HD]). Among these, DSC and ASD were designated as the primary outcomes, representing spatial overlap accuracy and boundary precision, respectively. The remaining contouring quality metrics, Volume, self-assessed competency scores, and interobserver variability (IOV) metrics — including standard deviation (SD), coefficient of variation (CV), and maximum-to-minimum volume ratio (MVR) — were treated as secondary outcomes. Correlation analyses between subjective and objective metrics, subregion-level analyses, and comparisons with automatic segmentation systems were classified as exploratory. Given the educational and exploratory nature, we prioritized primary outcomes (DSC, ASD); secondary/exploratory results were interpreted cautiously without formal multiplicity adjustment.

Correlation analyses were performed at two levels. At the regional level, self-rated proficiency scores for each subregion were correlated with objective metrics within that region, separately for pre-, post-, and delta (Δ) -change values. At the individual level, changes in subjective scores were correlated with changes in objective metrics. Spearman’s rank correlation was used throughout, with results visualized as heatmaps. To ensure consistent interpretation, objective metrics were directionally adjusted so that improvement in contouring quality (i.e., higher Inclusion, CI, DSC; lower RVD, ASD, DC, 95%HD) corresponds to positive correlation values (red), and deterioration to negative values (blue).

Analyses were performed using Python (version 3.10.8), and statistical significance was defined as a two-sided p-value < 0.05.

### Comparison with automatic segmentation systems

As an exploratory benchmark comparison, we evaluated two deep learning-based automatic segmentation systems (Medmind and United Imaging) using the same two clinical cases. Automatic contours were generated from the planning CT images and compared with the expert-defined reference volumes using the same contouring quality metrics as those applied to physician-generated delineations. Additional methodological details are provided in the Supplementary Materials.

## Results

### Improvements in self-assessed competency after training

In the training session, 27 radiation oncologists completed the questionnaire, among which 17 valid responses were retained after quality screening. As shown in Table 1, participants’ self-assessed competencies in cervical cancer CTV delineation improved significantly across all dimensions (*p* < 0.05). Confidence in independent contouring increased from 3.24 ± 0.83 to 4.12 ± 0.78 (Z = −2.950, *p* = 0.003), with 76.5% (13/17) rating themselves as “quite confident” or above post-training. Concern about making errors decreased (3.29 ± 0.92 to 4.00 ± 0.87; Z = −2.292, *p* = 0.022).

Knowledge-related scores also improved, including understanding of treatment principles (3.65 ± 0.79 to 4.29 ± 0.69; Z = −2.392, *p* = 0.017) and overall familiarity with CTV delineation (3.41 ± 0.94 to 4.12 ± 0.70; Z = −2.292, *p* = 0.022). Among anatomical regions, the most notable gains were in the uterine body (3.59 ± 1.00 to 4.65 ± 0.49; Z = −2.970) and cervical tumor with adjacent parametrial/vaginal areas (3.41 ± 1.00 to 4.53 ± 0.51; Z = −3.000), both with *p* = 0.003.

**Table 1:** Average self-reported scores before and after the education program

Item	Before the education program	After the education program	Z value	*p* value	Effect size *r*
Confidence in performing target delineation independently	3.24 ± 0.83 (2–5)	4.12 ± 0.78 (3–5)	−2.950	0.003*	0.716
Concern about errors during target delineation	3.29 ± 0.92 (1–5)	4.00 ± 0.87 (2–5)	−2.292	0.022*	0.556
Understanding of cervical cancer treatment principles	3.65 ± 0.79 (2–5)	4.29 ± 0.69 (3–5)	−2.392	0.017*	0.580
Familiarity with clinical target delineation for cervical cancer	3.41 ± 0.94 (1–5)	4.12 ± 0.70 (3–5)	−2.292	0.022*	0.556
Proficiency in delineating pelvic lymphatic drainage areas (internal iliac, obturator, common iliac, external iliac, presacral)	3.65 ± 0.93 (2–5)	4.53 ± 0.72 (3–5)	−2.765	0.006*	0.671
Proficiency in delineating para-aortic lymphatic drainage	3.53 ± 1.07 (1–5)	4.47 ± 0.72 (3–5)	−2.815	0.005*	0.683
Proficiency in delineating uterine body	3.59 ± 1.00 (2–5)	4.65 ± 0.49 (4–5)	−2.970	0.003*	0.720
Proficiency in delineating cervical tumor region and adjacent involved parametria/vaginal areas	3.41 ± 1.00 (2–5)	4.53 ± 0.51 (4–5)	−3.000	0.003*	0.728

Notes: Data are presented as mean ± SD (range). All items were rated on a 5-point Likert scale (1 = lowest level of confidence/proficiency, 5 = highest level; for the item on concern about errors, a higher score indicates less concern). p values were calculated using the Wilcoxon signed-rank test. * *p* < 0.05, indicating a statistically significant difference before and after the education program. Effect sizes were calculated as r = |Z|/√n, where n is the number of paired participants (*n* = 17)

### Improvements in CTV delineation accuracy and consistency

**Fig. 2:**
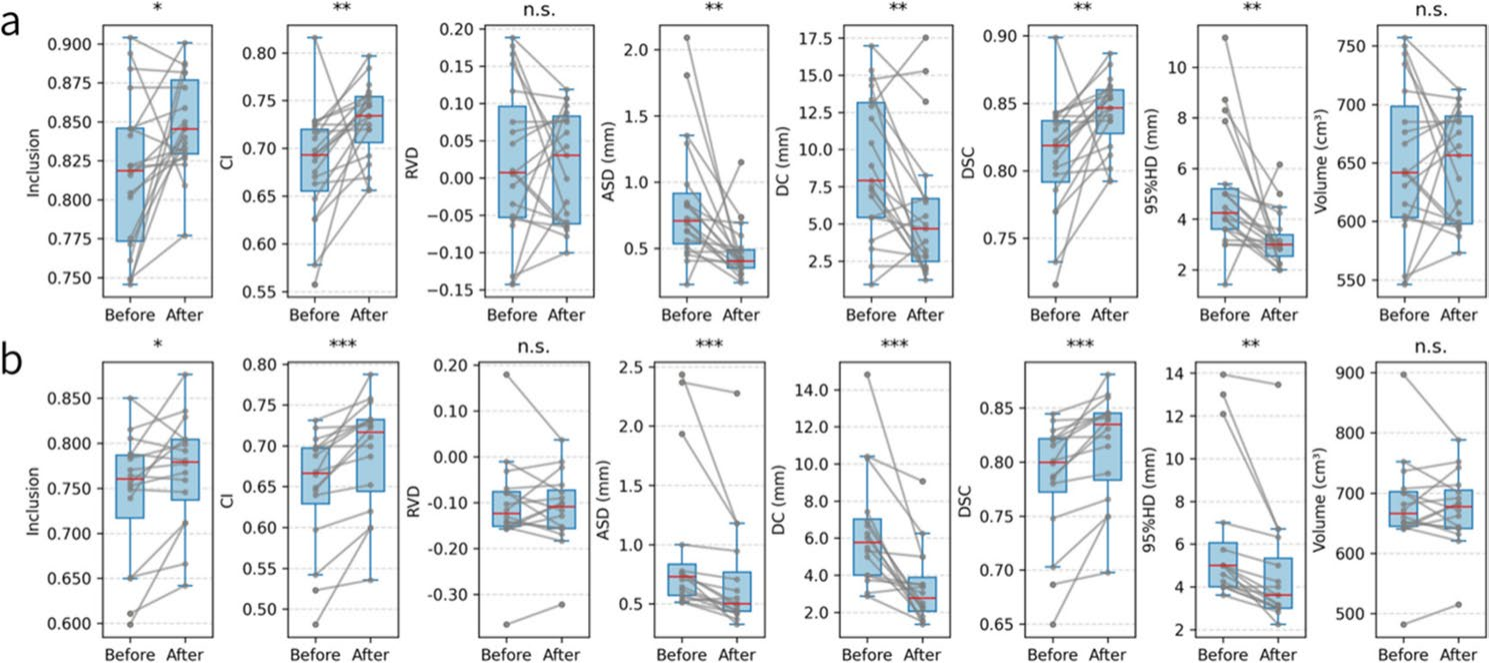
Comparison of contouring performance before and after training. **a** Results for Case 1 (definitive radiotherapy); **b** Results for Case 2 (postoperative radiotherapy). Metrics included Inclusion, CI, RVD, ASD, DC, DSC, 95%HD, and Volume, compared between pre- and post-training delineations against expert-defined reference volumes. Each dot represents an individual participant, with gray lines connecting paired pre- and post-training values. Blue boxplots represent the distribution of values, showing the median (red line), interquartile range (box), and range (whiskers). Statistical significance was assessed using paired t-tests or Wilcoxon signed-rank tests based on data distribution. Significance levels: *p* < 0.05 (*), *p* < 0.01 (**), *p* < 0.001 (***), n.s. = not significant

A total of 19 valid datasets were included for Case 1 (definitive radiotherapy) and 16 for Case 2 (postoperative radiotherapy). The education program significantly improved CTV delineation quality in both clinical scenarios (Fig. 2; Table 2).

In Case 1, post-training enhancements were observed in spatial agreement metrics, including DSC (0.81 ± 0.04 vs. 0.84 ± 0.03, *p* = 0.008), CI (0.68 ± 0.06 vs. 0.73 ± 0.04, *p* = 0.009), and Inclusion (0.82 ± 0.05 vs. 0.85 ± 0.03, *p* = 0.019). Geometric accuracy also improved, as reflected by reductions in ASD (*p* = 0.001), DC (*p* = 0.009), and 95%HD (*p* = 0.003). In Case 2, similar improvements were noted, with significantly higher DSC and CI (both *p* < 0.001) and lower ASD, DC, and 95%HD (all *p* < 0.01). Effect sizes for all significant outcomes ranged from medium to large (Table 2), indicating that the observed improvements were practically meaningful.

Although mean volume and RVD did not change significantly in either case, interobserver variability was markedly reduced following the intervention (Tables S2 and S3). In Case 1, larger decreases in SD, CV, and MVR suggested enhanced overall agreement, whereas in Case 2, more substantial reductions in Vmax and MVR reflected better control of extreme contouring deviations. These findings indicate that the educational program effectively improved both the accuracy and consistency of CTV delineation across different clinical contexts.

Training led to significant overall improvements in CTV delineation accuracy across key spatial metrics in Case 1. However, when analyzed by subregion (CTV-C, CTV-U, CTV-Na, CTV-Np), most metrics showed numerical gains without reaching statistical significance (Table S4). Notably, borderline improvements were observed in the para-aortic (CTV-Na) and pelvic nodal (CTV-Np) regions, suggesting potential value in region-specific training approaches.

**Table 2:** Comparison of CTV Parameters Before and After the Education Program in Case 1 and Case 2

Metric	Before the education program	After the education program	t/Z value	*P* value	Effect Size
1. Case 1					
Inclusion	0.82 ± 0.05 (0.75–0.90)	0.85 ± 0.03 (0.78–0.90)	−2.569	0.019*	d = 0.589
CI	0.68 ± 0.06 (0.56–0.82)	0.73 ± 0.04 (0.66–0.80)	−2.942	0.009*	d = 0.675
RVD	0.02 ± 0.11 (−0.14-0.19)	0.01 ± 0.08 (−0.10-0.12)	−0.644	0.777	*r* = 0.148
ASD(mm)	0.84 ± 0.48 (0.23–2.09)	0.46 ± 0.21 (0.24–1.15)	−3.139	0.001*	*r* = 0.720
DC(mm)	8.94 ± 4.81 (0.93–16.97)	5.85 ± 4.72 (1.25–17.53)	−2.535	0.009*	*r* = 0.582
DSC	0.81 ± 0.04 (0.72–0.90)	0.84 ± 0.03 (0.79–0.89)	−2.971	0.008*	d = 0.681
95%HD(mm)	5.00 ± 2.40 (1.41–11.18)	3.18 ± 1.10 (2.00–6.16)	−3.260	0.003*	*r* = 0.748
Volume(cm³)	649.77 ± 68.02 (545.96–757.37)	646.68 ± 48.43 (573.20–712.67)	−0.644	0.777	*r* = 0.148
2. Case 2					
Inclusion	0.74 ± 0.07 (0.60–0.85)	0.77 ± 0.06 (0.64–0.88)	−2.500	0.025*	d = 0.625
CI	0.65 ± 0.07 (0.48–0.73)	0.69 ± 0.07 (0.54–0.79)	−3.516	<0.001*	*r* = 0.879
RVD	−0.11 ± 0.11 (−0.37-0.18)	−0.11 ± 0.08 (−0.32-0.04)	−0.259	0.821	*r* = 0.065
ASD(mm)	0.97 ± 0.66 (0.51–2.43)	0.71 ± 0.50 (0.33–2.28)	−3.464	<0.001*	*r* = 0.866
DC(mm)	6.39 ± 3.17 (2.86–14.82)	3.38 ± 2.07 (1.34–9.07)	−3.361	<0.001*	*r* = 0.840
DSC	0.78 ± 0.06 (0.65–0.84)	0.81 ± 0.05 (0.70–0.88)	−3.516	<0.001*	*r* = 0.879
95%HD(mm)	6.26 ± 3.46 (3.61–13.93)	4.63 ± 2.75 (2.24–13.45)	−3.516	0.001*	*r* = 0.879
Volume(cm³)	678.97 ± 83.14 (481.95–896.48)	673.91 ± 63.08 (514.97–788.06)	−0.259	0.821	*r* = 0.065

Notes: Data are presented as mean ± SD (range). p values were calculated using paired-sample t-tests or Wilcoxon signed-rank tests, as appropriate * *p* < 0.05, indicating a statistically significant difference before and after the education program. Effect sizes were calculated as d = |t|/√n for paired t-tests and r = |Z|/√n for Wilcoxon signed-rank tests. Cohen’s effect size thresholds (small/medium/large): d (0.2/0.5/0.8) and r (0.1/0.3/0.5)

**Fig. 3:**
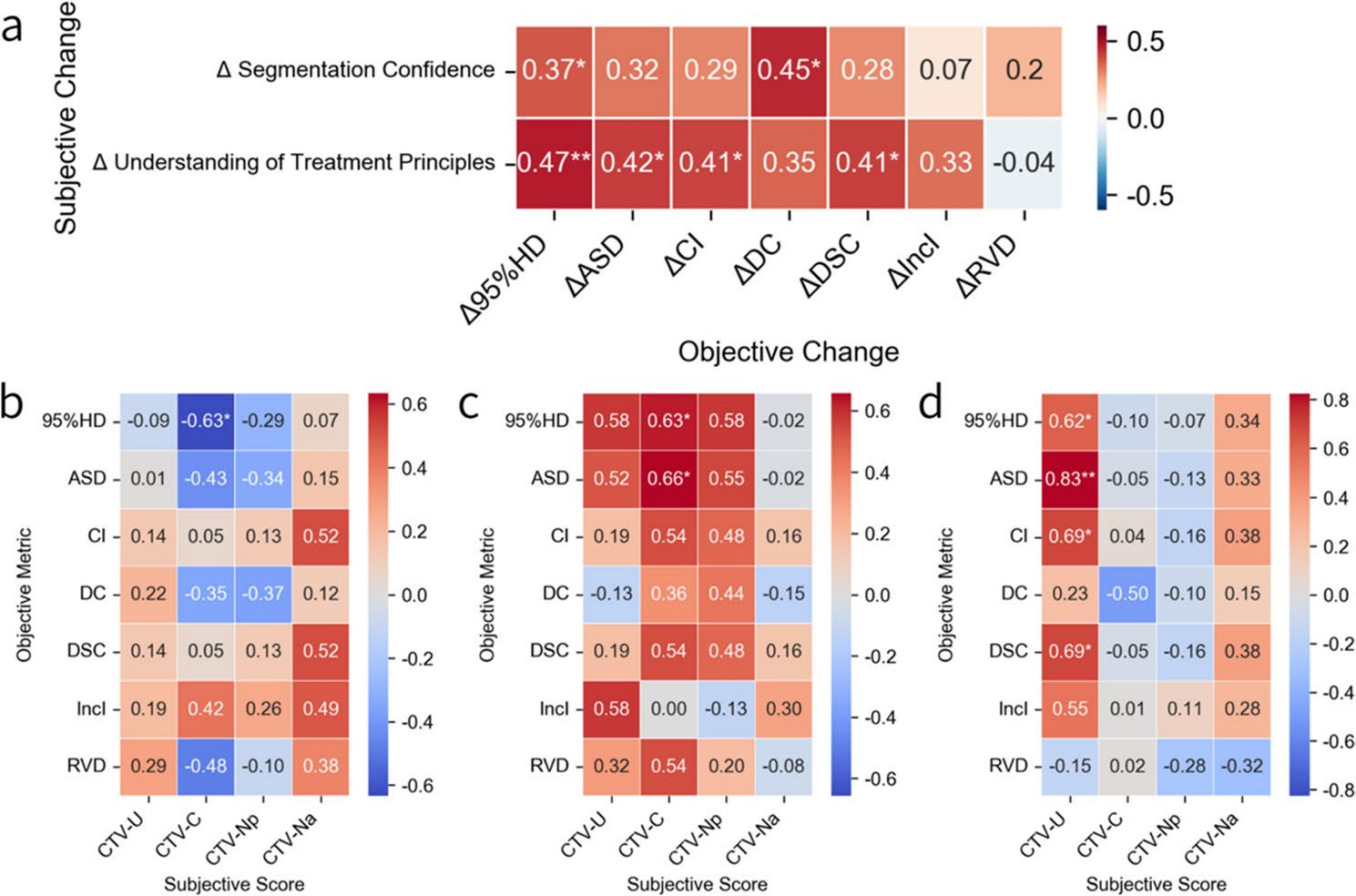
Spearman correlation heatmaps between subjective scores and objective contouring metrics. **a** Correlations between changes in subjective assessments (segmentation confidence and understanding of treatment principles) and changes in objective contouring metrics after training. (**b–d**) Correlations between subjective scores and objective metrics: (**b**) before training, (**c**) after training, and (**d**) score changes. The x-axis shows subjective scores for different CTV subregions (CTV-U, CTV-C, CTV-Np, CTV-Na), and the y-axis lists objective metrics (95%HD, ASD, CI, DC, DSC, Inclusion, RVD). For consistent interpretation, correlation coefficients of 95%HD, ASD, DC, and RVD were negated so that improvement in contour quality corresponds to positive correlations (red) and deterioration to negative correlations (blue). Color intensity reflects the strength of correlation. *p ≤ 0.05, **p ≤ 0.01

### Correlation between subjective improvements and contouring quality enhancements

As an exploratory analysis, we examined whether improvements in subjective self-assessment were associated with changes in objective contouring metrics. A total of 30 valid datasets (16 from Case 1 and 14 from Case 2) were combined for analysis. Improvements in subjective assessments following the educational intervention were positively correlated with changes in objective contouring metrics (Fig. 3a). Increased segmentation confidence was associated with better boundary precision and consistency (e.g., Δ95%HD: ρ = 0.37, *p* < 0.05; ΔDSC: ρ = 0.45, *p* < 0.05). Greater understanding of treatment principles showed stronger and more consistent correlations, particularly with Δ95%HD (ρ = 0.47, *p* < 0.01) and ΔDSC (ρ = 0.41, *p* < 0.05). These findings suggest that subjective gains—especially in conceptual understanding—may contribute to measurable improvements in contour accuracy and consistency.

### Region-specific alignment between self-assessment and contouring accuracy

A total of 11 valid datasets from Case 1 were included in this analysis. Before training, correlations between subjective scores and objective metrics were generally weak (Fig. 3b). A significant negative correlation was observed between CTV-C and 95%HD (*r* = − 0.63, *p* ≤ 0.05), suggesting limited alignment between perceived and actual contour quality. Notably, for the para-aortic lymph node drainage region—an anatomically complex area—participants tended to rate their performance conservatively, and the objective metrics also reflected poor contour quality.

Following training, stronger positive correlations emerged (Fig. 3c), particularly in the cervix and adjacent pelvic regions (CTV-C: ASD *r* = 0.66, *p* ≤ 0.05; 95%HD *r* = 0.63, *p* ≤ 0.05), suggesting improved self-awareness of delineation performance after instruction. In the analysis of pre- to post-training changes (Fig. 3d), the uterine body showed the strongest associations between subjective and objective improvements (ASD: *r* = − 0.83, *p* ≤ 0.01; CI: *r* = 0.69, DSC: *r* = 0.69, both *p* ≤ 0.05), likely due to its clearer anatomical boundaries. No significant associations were observed in other regions.

### Comparison of automatic segmentation systems and manual delineation performance

**Fig. 4:**
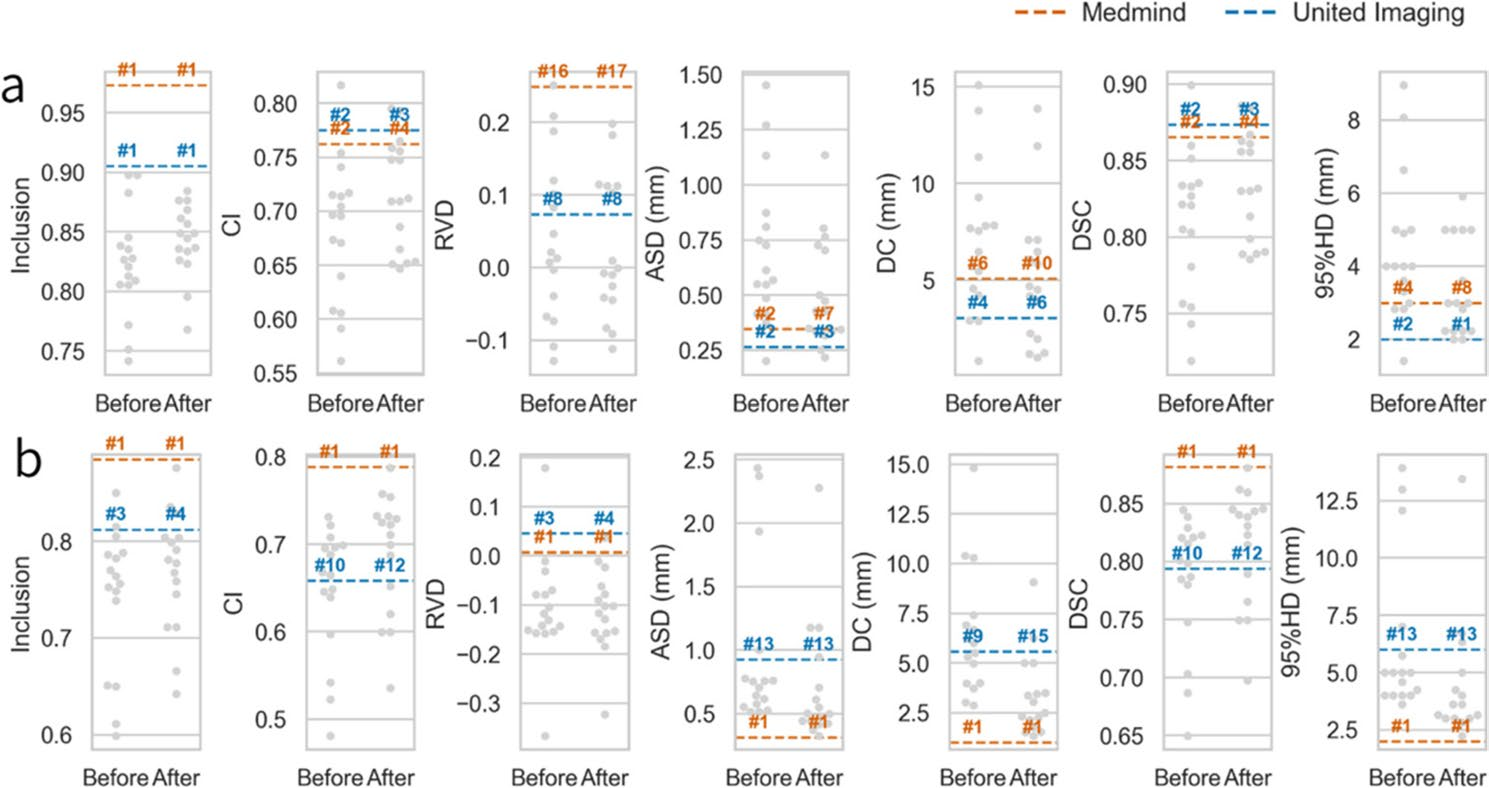
Comparison of automatic segmentation systems (Medmind and United Imaging) with physician manual delineations before and after training. **a** Results for Case 1 (definitive radiotherapy); **b** Results for Case 2 (postoperative radiotherapy). Metrics included Inclusion, CI, RVD, ASD, DC, DSC, and 95%HD. Grey dots represent individual physicians’ delineation performances before and after training, with swarm plots illustrating distribution. Dashed lines indicate the performance of two automatic segmentation systems: Medmind (orange) and United Imaging (blue), with corresponding ranks labeled above each line

In an exploratory comparison, both automatic segmentation systems demonstrated high and consistent performance across spatial and geometric metrics (Fig. 4). Following training, physicians’ delineations showed marked improvements in spatial overlap and boundary accuracy, shifting closer to the automated benchmarks and exhibiting reduced variability. These findings suggest that structured training substantially narrowed the performance gap between clinicians and automated systems. Detailed system-specific performance and rankings are provided in the Supplementary Materials.

## Discussion

Our study demonstrates that a structured educational program significantly enhances the precision and consistency of CTV delineation among radiation oncologists in cervical cancer radiotherapy. Participants showed marked improvements in self-assessed confidence and proficiency, alongside objective gains in delineation accuracy. This finding is consistent with studies in other tumor sites, such as rectal cancer, where structured education improved delineation accuracy [16].

To our knowledge, this is the first study to systematically evaluate a structured educational program targeting CTV delineation in cervical cancer EBRT using both subjective and objective metrics. We integrated a comprehensive set of spatial evaluation metrics — with DSC and ASD as primary outcomes — alongside secondary measures (CI, DC, 95%HD, Inclusion, RVD) and explored their correlation with self-assessed competency, offering a novel perspective on how subjective improvements may translate into measurable gains in contouring accuracy.

The participants in our study were all practicing radiation oncologists from tertiary-level medical centers across China, with a balanced representation of junior and senior professionals and an average of over a decade of clinical experience in gynecologic oncology. Despite this, baseline delineations revealed substantial interobserver variability. In Case 2, for example, CTV volumes varied by as much as 1.86-fold across participants (range: 481.95–896.48 cm³). This finding is in line with previous studies indicating that even experienced radiation oncologists exhibit significant variation in contouring complex pelvic anatomy [4]. Moreover, significant variability persists even among practitioners with prior contouring training [17]. This highlights the necessity of structured educational interventions, even for experienced practitioners. Improved delineation accuracy has also been linked to better local control and reduced toxicity in clinical studies of cervical cancer radiotherapy, underscoring the clinical value of standardization efforts [18]. In addition, such educational interventions may facilitate the standardization of experimental protocols in future multi-center translational or clinical research, thereby enhancing data comparability and collaborative efficiency.

Interestingly, while overall delineation quality improved significantly, subregion-specific analysis revealed variability in the magnitude of improvement. Regions such as the para-aortic lymph nodes and pelvic nodal areas demonstrated borderline statistical significance for spatial accuracy improvements. This finding indicates that anatomically complex regions might benefit from additional targeted educational efforts. Future training programs should consider incorporating specialized modules focusing specifically on these challenging regions to enhance precision further.

Our exploratory correlation analyses offer additional insights, suggesting that subjective improvements in understanding and confidence directly translate into objective contouring accuracy. After training, a strengthened correlation was observed between self-assessed proficiency and measured delineation quality. This indicates improved self-awareness among participants regarding their delineation competencies, which is critical for ongoing professional development. Improved self-awareness of technical competence is a recognized benefit of structured feedback-based training in clinical education [16].

In theory, subjective ratings should positively correlate with objective contouring quality—better self-assessed anatomical knowledge should translate into more accurate delineations. However, at the pre-training stage, the correlations were mixed, with nearly equal proportions of positive and negative relationships. This likely reflects limited self-assessment accuracy prior to structured feedback, especially in tasks involving spatial reasoning and visual judgment [19]. Additionally, variations in individual contouring styles (e.g., delineating larger or smaller ROIs) likely contributed to these inconsistencies. Interestingly, for complex regions such as the para-aortic lymphatic drainage area, both low self-assessment and suboptimal contouring quality were observed, yet the correlation between the two remained positive—suggesting that participants were relatively aware of their limitations even in difficult areas. Notably, changes in subjective ratings for the uterine body from pre- to post-training closely mirrored objective improvements, likely due to its well-defined boundaries and consistent recognition among participants. In contrast, the perception of quality changes in other regions remained ambiguous. At the subregion level, correlation patterns were heterogeneous and should be interpreted cautiously. Some positive associations were observed after training, particularly in anatomically clearer regions, but these findings were exploratory and not consistently observed across all regions.

As an exploratory benchmark comparison, the two automatic segmentation systems demonstrated generally high contouring performance. Following training, physician delineations moved closer to these automated benchmarks across several key metrics, suggesting that structured education may help narrow the gap between clinician performance and AI-assisted contouring. However, the inability of one system to generate CTV-Na and the observed volumetric deviations indicate that automated tools are not uniformly reliable and still require careful human oversight [20]. Our results suggest that educational interventions are not made obsolete by AI; rather, they are essential for training clinicians to a level where they can effectively supervise, validate, and refine automated outputs [21]. This supports a human-in-the-loop approach, combining AI efficiency with expert clinical judgment to improve patient outcomes.

Despite the encouraging improvements, this study has several limitations. First, participation in the tasks was voluntary. The final analytical cohorts were limited to those with complete, high-quality paired data, which reduced the sample size compared to the initial enrollment. This may introduce selection bias, as participants who completed all components might be more motivated or specialized, potentially limiting the generalizability of our findings. Second, the pre- and post-training delineations were performed on the same CT datasets. Although this design enabled a controlled within-participant comparison, it may also have introduced a case-familiarity effect; thus, part of the observed improvement may reflect recall rather than the educational intervention alone. Third, only two representative clinical scenarios were included, namely one definitive radiotherapy case and one postoperative radiotherapy case. Although these cases were selected to facilitate in-depth discussion within a limited training timeframe, they do not capture the full anatomical and clinical variability encountered in routine practice, which may limit the generalizability of the findings. Fourth, the assessment was limited to the immediate post-training period; therefore, the long-term retention of contouring skills and their transferability to new clinical cases remain unknown. Future studies should incorporate a broader range of clinical scenarios, including new post-training test cases, as well as delayed follow-up assessments to better evaluate the transferability and long-term retention of the intervention effects. Furthermore, anatomically complex regions such as the para-aortic and pelvic nodal areas showed only borderline improvement, suggesting that future programs may benefit from region-specific training modules.

Although this study did not directly evaluate dosimetric outcomes, the observed geometric improvements may still have clinical relevance. Prior research in pelvic radiotherapy has shown that even small variations in CTV delineation can lead to meaningful differences in target coverage and sparing of organs at risk [22]. In our study, the reductions in geometric distance metrics (ASD and 95%HD) and the improvements in overlap indices (DSC and CI) suggest that training-related contouring refinements may potentially translate into more precise dose distributions. Furthermore, the decreased interobserver variability indicates improved treatment consistency, which is important for reproducible clinical practice [12]. However, because we did not perform plan-level dosimetric comparisons, the dosimetric and clinical impact of these geometric improvements cannot be directly inferred. Future studies incorporating plan-level dosimetric comparisons and long-term clinical endpoints are warranted to further evaluate these downstream effects.

In conclusion, our findings strongly support structured educational programs to enhance CTV delineation accuracy and consistency in cervical cancer radiotherapy. Targeted training interventions not only reduce interobserver variability but also positively impact clinicians’ confidence and accuracy, potentially translating into improved clinical outcomes. Further research should explore long-term retention, applicability across diverse clinical settings, and integration into broader educational curricula.

## Supplementary Information

Supplementary Material 1.

## Data Availability

The datasets used and/or analysed during the current study are available from the corresponding author on reasonable request.
