# Supplementary material for: Enhancing the precision and uniformity of clinical target volume delineation for cervical cancer through a structured educational program
Source: BMC Med Educ. 2026 Apr 13;26:856. doi: 10.1186/s12909-026-09203-w (PMC13217681; doi:10.1186/s12909-026-09203-w)
Supplement: Supplementary file 1 — Supplementary Material 1. [file 12909_2026_9203_MOESM1_ESM.docx]

**Supplementary Materials**

**• Supplementary** **Methods**

**• Supplementary Results**

**• Supplementary Tables**

**• Questionnaire**

**Supplementary Methods**

**Comparison with Automatic Segmentation Systems**

As an exploratory benchmark comparison, we evaluated two deep learning-based automatic segmentation systems, Medmind (Beijing MedMind Technology Co., Ltd.) and United Imaging (Shanghai United Imaging Healthcare Co., Ltd.), using the same two clinical cases. For both cases, automatic contours were generated from planning CT images and clinical information and were evaluated against the expert-defined reference volumes using the same contouring quality metrics as those applied to physician-generated delineations.

System performances were shown as dashed lines in the figures, with rankings labeled above each line. Rankings reflected metric-specific optimal values: higher values were better for Inclusion, CI, and DSC; lower values were better for ASD, DC, and 95%HD; and for RVD, ranks were assigned based on proximity to zero.

For Case 2, both automatic systems generated full CTV contours and were compared directly with physician-delineated CTVs. For Case 1, because United Imaging was unable to generate CTV-Na, both automatic systems were compared with the physicians’ CTV_S, defined as the combined volume of CTV-Np, CTV-U, and CTV-C.

**Supplementary Results**

**Comparison of Automatic Segmentation Systems and Manual Delineation Performance**

The performance of two automatic segmentation systems (Medmind and United Imaging) was compared with physician manual delineations before and after training in both cases (Figure 4).

For Case 1, both automatic systems were evaluated against the physicians’ CTV_S because United Imaging was unable to generate the para-aortic nodal volume (CTV-Na) (Figure 4a). Overall, both systems demonstrated favorable spatial overlap and geometric accuracy, although RVD values were less favorable, particularly for Medmind, indicating larger volume deviations. For Case 2, both systems were compared with the physicians’ full CTV delineations (Figure 4b). Medmind generally ranked highest across the evaluated metrics, whereas United Imaging performed comparatively well for Inclusion and RVD but less favorably for several other geometric measures.

For both cases, physicians’ delineations after training showed marked improvements in spatial overlap (Inclusion, CI, DSC) and boundary accuracy (ASD, DC, 95%HD), shifting closer to the automatic systems’ performance levels and exhibiting reduced variability. The figure clearly illustrates that, although automatic segmentation systems achieved high and consistent performance, structured training enabled physicians to substantially improve and approach these automated benchmarks in multiple metrics. These supplementary findings provide additional benchmark context for the main results reported in the manuscript.

**Supplementary Tables**

**Table S1. Definitions and Classification of Contouring Quality Metrics**

| **Category** | **Metric** | **Definition** |
| --- | --- | --- |
| **1. Contouring Quality Metrics** | | |
| Spatial overlap and consistency metrics | Dice Similarity Coefficient (DSC) | DSC = 2 × \|A ∩ B\| / (\|A\| + \|B\|): Overlap between delineated and reference volumes. |
|  | Conformity Index (CI) | CI = V_overlap_ / V_union_: Overlap volume divided by total combined volume. |
|  | Inclusion Index (Inclusion) | Inclusion = V_overlap_ / V_ref_: Proportion of reference volume covered. |
|  | Relative Volume Difference (RVD) | RVD = (V_test_ - V_ref_) / V_ref_: Relative volume bias. |
| Geometric boundary accuracy metrics | 95th percentile Hausdorff Distance (95%HD) | Surface distance excluding the top 5% of outliers. |
|  | Average Surface Distance (ASD) | Mean distance between corresponding surface points of the two volumes. |
|  | Distance between Centers of Mass (DC) | Distance between the centroids of the delineated and reference volumes. |
| **2. Interobserver Variability Metrics (IOV)** | | |
|  | Standard Deviation (SD) | Dispersion of delineated volumes across participants. |
|  | Coefficient of Variation (CV) | CV = SD / Mean × 100%: Normalized variability. |
|  | Max-to-Min Volume Ratio (MVR) | MVR = V_max_ / V_min_: Ratio of the largest to the smallest delineated volume. |

**Table S2. Comparative Analysis of Target Volume Variability Metrics Before and After Educational Intervention in Case 1**

|  | Vmax (cm³) | Vmin (cm³) | Mean (cm³) | SD (cm³) | MVR | CV(%) |
| --- | --- | --- | --- | --- | --- | --- |
| Before the education program | 757.37 | 545.96 | 649.77 | 68.02 | 1.39 | 10.47 |
| After the education program | 712.67 | 573.20 | 646.68 | 48.43 | 1.24 | 7.49 |
| Decrease ratio | 5.90% | -4.99% | 0.48% | 28.79% | 10.37% | 28.45% |

**Table S3. Comparative Analysis of Target Volume Variability Metrics Before and After Educational Intervention in Case 2**

|  | Vmax (cm³) | Vmin (cm³) | Mean (cm³) | SD (cm³) | MVR | CV(%) |
| --- | --- | --- | --- | --- | --- | --- |
| Before the education program | 896.48 | 481.95 | 678.97 | 83.14 | 1.86 | 12.24 |
| After the education program | 788.06 | 514.97 | 673.91 | 63.08 | 1.53 | 9.36 |
| Decrease ratio | 12.09% | -6.85% | 0.75% | 24.12% | 17.73% | 23.55% |

**Table S4. Comparison of Contouring Metrics Before and After the Education Program Across Different CTV Sub-Regions in Case 1**

| CTV Region | Metric | Before the education program | After the education program | t/Z value | P value |
| --- | --- | --- | --- | --- | --- |
| CTV | Inclusion | 0.82±0.05 (0.75-0.90) | 0.85±0.03 (0.78-0.90) | -2.569 | 0.019* |
| CTV | CI | 0.68±0.06 (0.56-0.82) | 0.73±0.04 (0.66-0.80) | -2.942 | 0.009* |
| CTV | RVD | 0.02±0.11 (-0.14-0.19) | 0.01±0.08 (-0.10-0.12) | -0.644 | 0.777 |
| CTV | ASD(mm) | 0.84±0.48 (0.23-2.09) | 0.46±0.21 (0.24-1.15) | -3.139 | 0.001* |
| CTV | DC(mm) | 8.94±4.81 (0.93-16.97) | 5.85±4.72 (1.25-17.53) | -2.535 | 0.009* |
| CTV | DSC | 0.81±0.04 (0.72-0.90) | 0.84±0.03 (0.79-0.89) | -2.971 | 0.008* |
| CTV | 95%HD(mm) | 5.00±2.40 (1.41-11.18) | 3.18±1.10 (2.00-6.16) | -3.260 | 0.003* |
| CTV | Volume | 649.77±68.02 (545.96-757.37) | 646.68±48.43 (573.20-712.67) | -0.644 | 0.777 |
| CTV-C | Inclusion | 0.79±0.14 (0.50-1.00) | 0.81±0.11 (0.59-1.00) | -0.811 | 0.429 |
| CTV-C | CI | 0.52±0.11 (0.36-0.76) | 0.57±0.09 (0.44-0.75) | -1.497 | 0.153 |
| CTV-C | RVD | 0.35±0.48 (-0.39-1.23) | 0.27±0.39 (-0.35-1.23) | 1.220 | 0.239 |
| CTV-C | ASD(mm) | 1.57±0.81 (0.34-3.31) | 1.15±0.58 (0.35-2.74) | 1.631 | 0.121 |
| CTV-C | DC(mm) | 6.03±3.88 (1.74-14.92) | 5.43±3.57 (0.85-16.15) | -0.806 | 0.959 |
| CTV-C | DSC | 0.68±0.09 (0.53-0.87) | 0.72±0.07 (0.62-0.86) | -1.596 | 0.129 |
| CTV-C | 95%HD(mm) | 9.64±4.95 (2.83-22.00) | 7.44±3.59 (2.83-16.00) | -2.896 | 0.035* |
| CTV-C | Volume | 136.71±48.85 (61.49-225.76) | 128.76±39.57 (65.73-225.72) | 1.217 | 0.240 |
| CTV-U | Inclusion | 0.71±0.12 (0.53-0.92) | 0.76±0.10 (0.58-0.90) | -1.907 | 0.073 |
| CTV-U | CI | 0.63±0.10 (0.45-0.87) | 0.64±0.10 (0.45-0.83) | -0.793 | 0.438 |
| CTV-U | RVD | -0.13±0.33 (-0.44-0.90) | -0.02±0.34 (-0.41-0.89) | -3.139 | 0.026* |
| CTV-U | ASD(mm) | 1.52±0.84 (0.12-4.23) | 1.20±0.93 (0.18-4.24) | -2.656 | 0.140 |
| CTV-U | DC(mm) | 4.73±3.39 (0.76-15.89) | 5.03±3.68 (0.70-15.80) | -1.932 | 0.730 |
| CTV-U | DSC | 0.77±0.07 (0.62-0.93) | 0.78±0.08 (0.62-0.91) | -0.819 | 0.424 |
| CTV-U | 95%HD(mm) | 11.78±5.91 (1.00-26.00) | 8.89±6.28 (1.00-26.00) | 1.916 | 0.071 |
| CTV-U | Volume | 73.15±27.88 (47.19-158.46) | 81.93±28.75 (49.67-158.42) | -3.139 | 0.026* |
| CTV-Na | Inclusion | 0.74±0.16 (0.36-0.94) | 0.79±0.11 (0.56-0.95) | -1.731 | 0.103 |
| CTV-Na | CI | 0.62±0.13 (0.36-0.80) | 0.68±0.09 (0.46-0.82) | -2.107 | 0.051 |
| CTV-Na | RVD | -0.06±0.22 (-0.64-0.25) | -0.04±0.17 (-0.25-0.32) | -0.512 | 0.616 |
| CTV-Na | ASD(mm) | 1.63±2.02 (0.22-7.97) | 0.80±0.74 (0.19-2.77) | -2.391 | 0.030* |
| CTV-Na | DC(mm) | 8.82±7.42 (2.26-31.33) | 5.99±4.16 (2.26-16.55) | -2.059 | 0.070 |
| CTV-Na | DSC | 0.76±0.11 (0.53-0.89) | 0.81±0.06 (0.63-0.90) | -2.296 | 0.039* |
| CTV-Na | 95%HD(mm) | 11.20±12.71 (2.00-49.00) | 5.73±5.59 (1.41-24.00) | -2.627 | 0.048* |
| CTV-Na | Volume | 90.75±21.07 (35.01-121.02) | 93.30±16.33 (72.85-127.49) | -0.512 | 0.616 |
| CTV-Np | Inclusion | 0.79±0.07 (0.64-0.91) | 0.81±0.04 (0.72-0.87) | -1.365 | 0.191 |
| CTV-Np | CI | 0.65±0.09 (0.46-0.78) | 0.69±0.06 (0.49-0.76) | -2.959 | 0.009* |
| CTV-Np | RVD | 0.02±0.26 (-0.30-0.81) | -0.01±0.15 (-0.17-0.54) | -1.112 | 0.691 |
| CTV-Np | ASD(mm) | 0.99±1.02 (0.24-3.73) | 0.71±0.85 (0.27-3.86) | -2.201 | 0.088* |
| CTV-Np | DC(mm) | 6.75±5.99 (0.88-22.47) | 4.77±5.56 (0.78-25.40) | -2.154 | 0.100 |
| CTV-Np | DSC | 0.78±0.07 (0.63-0.88) | 0.81±0.05 (0.66-0.86) | -3.006 | 0.008* |
| CTV-Np | 95%HD(mm) | 6.95±8.45 (2.00-30.17) | 4.85±6.72 (2.00-30.64) | -2.817 | 0.026* |
| CTV-Np | Volume | 380.61±96.88 (261.58-675.78) | 368.41±57.33 (309.04-572.04) | -1.112 | 0.691 |

Notes: Data are presented as mean ± SD (range). p values were calculated paired-sample t-tests or Wilcoxon signed-rank tests, as appropriate. * p < 0.05, indicating a statistically significant difference before and after the education program.

**Questionnaire**

**Cervical Cancer External Beam Target Delineation Course Evaluation**

**Before the Course**

**Your Name:** [Fill-in-the-blank] *

**Your Gender:** [Single choice] *

○ Male　○ Female

**Your Age:** [Fill-in-the-blank] *

**Your Professional Title:** [Single choice] *

○ Chief Physician

○ Associate Chief Physician

○ Attending Physician

○ Resident Physician

**Years of Work Experience:** [Single choice] *

○ 0–1 year

○ 1–5 years

○ 5–10 years

○ 10–15 years

○ More than 15 years

**How many gynecological radiotherapy patients have you treated so far?** [Single choice] *

○ 0–10

○ 10–25

○ 25–50

○ 50–100

○ More than 100

**How confident are you in independently completing target delineation?** [Single choice] *

○ Not confident at all

○ Slightly confident

○ Moderately confident

○ Quite confident

○ Very confident

**Do you feel worried when performing target delineation?** [Single choice] *

○ Often worried about making mistakes or not meeting standards

○ Somewhat worried

○ Occasionally worried

○ Rarely worried

○ Calm, not worried at all

**How well do you understand the principles of cervical cancer treatment?** [Single choice] *

○ Not at all

○ Slightly understand

○ Basically understand

○ Relatively proficient

○ Fully proficient

**How familiar are you with target delineation in real clinical cervical cancer cases?** [Single choice] *

○ Completely unfamiliar

○ Slightly familiar

○ Basically familiar

○ Relatively familiar

○ Very familiar

**How well do you grasp the delineation boundaries of the following anatomical structures?** [Matrix single choice] *

|  | **Not at all** | **Slightly understand** | **Basically understand** | **Relatively proficient** | **Fully proficient** |
| --- | --- | --- | --- | --- | --- |
| Pelvic lymphatic drainage areas (internal iliac, obturator, common iliac, external iliac, presacral) | ○ | ○ | ○ | ○ | ○ |
| Para-aortic lymphatic drainage | ○ | ○ | ○ | ○ | ○ |
| Uterine body | ○ | ○ | ○ | ○ | ○ |
| Cervical tumor region and adjacent involved parametrial and vaginal areas | ○ | ○ | ○ | ○ | ○ |

**After the Course**

**After completing the course, how confident are you in independently completing target delineation?** [Single choice] *

○ Not confident at all

○ Slightly confident

○ Moderately confident

○ Quite confident

○ Very confident

**After completing the course, do you feel worried when performing target delineation?** [Single choice] *

○ Often worried about making mistakes or not meeting standards

○ Somewhat worried

○ Occasionally worried

○ Rarely worried

○ Calm, not worried at all

**After completing the course, how well do you understand the principles of cervical cancer treatment?** [Single choice] *

○ Not at all

○ Slightly understand

○ Basically understand

○ Relatively proficient

○ Fully proficient

**After completing the course, how familiar are you with target delineation in real clinical cervical cancer cases?** [Single choice] *

○ Completely unfamiliar

○ Slightly familiar

○ Basically familiar

○ Relatively familiar

○ Very familiar

**After completing the course, how well do you grasp the delineation boundaries of the following anatomical structures?** [Matrix single choice] *

|  | **Not at all** | **Slightly understand** | **Basically understand** | **Relatively proficient** | **Fully proficient** |
| --- | --- | --- | --- | --- | --- |
| Pelvic lymphatic drainage areas (internal iliac, obturator, common iliac, external iliac, presacral) | ○ | ○ | ○ | ○ | ○ |
| Para-aortic lymphatic drainage | ○ | ○ | ○ | ○ | ○ |
| Uterine body | ○ | ○ | ○ | ○ | ○ |
| Cervical tumor region and adjacent involved parametrial and vaginal areas | ○ | ○ | ○ | ○ | ○ |

**How do you find the difficulty level of the course content?** [Single choice] *

○ Very easy

○ Relatively easy

○ Moderate

○ Relatively difficult

○ Very difficult

**How useful do you think this course is for improving external beam target delineation in cervical cancer?** [Single choice] *

○ Not useful at all

○ Almost not useful

○ Neutral

○ Quite useful

○ Very useful

**How helpful were the interactive components of the course (e.g., group discussions, case analyses) for your learning?** [Single choice] *

○ Not helpful at all

○ Almost not helpful

○ Neutral

○ Quite helpful

○ Very helpful

**Would you be willing to recommend this course to your colleagues?** [Single choice] *

○ Definitely not recommend

○ Not recommend much

○ Neutral

○ Quite recommend

○ Strongly recommend

**How satisfied are you with the course overall?** [Single choice] *

○ Very dissatisfied

○ Dissatisfied

○ Neutral

○ Quite satisfied

○ Very satisfied

**How would you rate the teaching ability of the course instructor(s)?** [Single choice] *

○ Very poor

○ Poor

○ Neutral

○ Good

○ Very good

**Are you satisfied with the organization and arrangement of the course?** [Single choice] *

○ Very dissatisfied

○ Dissatisfied

○ Neutral

○ Quite satisfied

○ Very satisfied

**Do you have any suggestions or feedback about the course?** [Fill-in-the-blank]
